# Assessment of peri- and postoperative complications and Karnofsky-performance status in head and neck cancer patients after radiation or chemoradiation that underwent surgery with regional or free-flap reconstruction for salvage, palliation, or to improve function

**DOI:** 10.1186/1748-717X-6-109

**Published:** 2011-09-06

**Authors:** Christian Simon, Cem Bulut, Philippe A Federspil, Marc W Münter, Katja Lindel, Zazie Bergmann, Serkan Sertel, Sarah Leitzbach, Peter K Plinkert

**Affiliations:** 1 University of Heidelberg, Department of Otolaryngology - Head and Neck Surgery, Im Neuenheimer Feld 400, 69120 Heidelberg, Germany; 2 University of Heidelberg, Department of Radiation Oncology, Im Neuenheimer Feld 400, 69120 Heidelberg, Germany

**Keywords:** head and neck cancer, radiation, free flap, regional flap, Karnofsky performance status

## Abstract

**Background:**

Surgery after (chemo)radiation (RCTX/RTX) is felt to be plagued with a high incidence of wound healing complications reported to be as high as 70%. The additional use of vascularized flaps may help to decrease this high rate of complications. Therefore, we examined within a retrospective single-institutional study the peri--and postoperative complications in patients who underwent surgery for salvage, palliation or functional rehabilitation after (chemo)radiation with regional and free flaps. As a second study end point the Karnofsky performance status (KPS) was determined preoperatively and 3 months postoperatively to assess the impact of such extensive procedures on the overall performance status of this heavily pretreated patient population.

**Findings:**

21 patients were treated between 2005 and 2010 in a single institution (17 male, 4 female) for salvage (10/21), palliation (4/21), or functional rehabilitation (7/21). Overall 23 flaps were performed of which 8 were free flaps. Major recipient site complications were observed in only 4 pts. (19%) (1 postoperative haemorrhage, 1 partial flap loss, 2 fistulas) and major donor site complications in 1 pt (wound dehiscence). Also 2 minor donor site complications were observed. The overall complication rate was 33%. There was no free flap loss. Assessment of pre- and postoperative KPS revealed improvement in 13 out of 21 patients (62%). A decline of KPS was noted in only one patient.

**Conclusions:**

We conclude that within this (chemo)radiated patient population surgical interventions for salvage, palliation or improve function can be safely performed once vascularised grafts are used.

## Findings

Surgery after (chemo)radiation (RCTX/RTX) therapy is felt to be plagued with a high incidence of wound complications as the consequence of radiation induced wound bed changes [1]. Major peri- and postoperative complications upon surgery after RCTX or RTX are reported to be up to 73% for i.e. salvage laryngectomies [2]. The use of regional and free tissue transfer appears to decrease these complications. However studies on the incidence of major peri-and postoperative complications after procedures that include using vascularized tissue transfer still display highly variable rates that range between 10% [3] and 66% (for doubly irradiated patients) [4]. For salvage laryngectomies with reinforcement of the pharyngeal closure using vascularized tissue transfer the incidence of fistula formation is reported to be between 18% [5] and 29% [6] and there is still debate whether or not flaps help to decrease the incidence of such fistulas [7-10]. Thus, there remains a question on the safety of performing surgical procedures on (chemo)radiated patients and the role of vascularized tissue transfer within this patient population.

We therefore undertook a retrospective chart review on our own patient population in order to assess the incidence of peri- and postoperative complications after procedures including free flap and/or regional flap reconstruction in (chemo)radiated patients undergoing surgical salvage for recurrent disease, surgical functional rehabilitation or palliation. Given that such extensive procedures as free and regional flaps may compromise the performance status of the patients, we added an assessment of the pre- and postoperative Karnofsky performance status (KPS) as a second endpoint to this study.

All patients were treated at the University of Heidelberg Medical Center between 2005 and 2010. All (chemo)radiated patients that received a vascularized transplant within this interval and were operated on by the authors C.S. and P. A. F. The institutional review board at UHMC approved this retrospective analysis and the study has therefore been performed in accordance with the ethical standards laid down in the 1964 declaration of Helsinki. All patients gave their informed consent prior to their inclusion in the study. Surgical intervention was chosen as per the treating physician discretion. Free flap reconstruction was used on the basis of the surgeon's preference (Table 1).

**Table 1:** Patients characteristics and treatment categories: Treatment categories are divided into salvage and palliative procedures, procedures to improve function, closure of a fistula, and management of a radiation induced wound healing complication.

Number	Age	Diagnosis	Gender	Treatment category
1	59	OC (T2N0M0) 98, Hypopharynx-/Larynx(T4N2cM0) 10/06, Hypoharynx-/Larynx recurrence 11/07	male	salvage
2	59	OC 97, Hypopharynx (T2N1M0) 04, hypopharynx recurrence 9/07	male	fistula
3	56	oropharynx (T3N2bM0) 03/07, oropharynx recurrence 12/07	male	salvage
4	47	OC (T2N1M0) 02/06, oropharynx 03/07	male	salvage
5	79	Ear SSC 04/04 (T1N3M0), SCC recurrence with involvement of temporal bone, parotid gland 12/05	male	palliative
6	72	SCC temple region (T4N0M0) 08/06, recurrence 11/07	male	palliative
7	62	Ear basosquamous CC (T4N0M0) 01/01, recurrence with cerebral infiltration 03/06	male	palliative
8	66	Larynx-SCC (T4N0M0) 97, regional recurrence 06	male	palliative
9	60	Hypopharynx-SCC (T3N1M0) 02/94	male	functional
10	52	CUP-Syndrom (T0N2bM0) 85, Oropharynx SCC (T4N0M0) 11/04	male	salvage
11	56	CUP-Syndrom (T0N2bM0) 85, Oropharynx SCC (T4N0M0) 11/04, Larynx SCC (T4N0M0) 12/07	male	salvage
12	58	Oropharynx SCC (T3N0M0) 11/04, Hypopharynx SCC (T4N0M0) 08/06, Rektumkarzinom (T3N2M1) 09/07	female	salvage
13	66	Hypopharynx SCC (T1N2M0) 07/06	female	functional
14	48	Larynx SCC (T4N2M0) 06/07	male	postradiation wound healing complication
15	64	Nose SCC 05/08 (T2N0M0), recurrence (T4N0M0) 01/09, recurrence (T4N0M0) 07/09	male	salvage
16	55	Oropharynx SCC (T2N2BM0) 05/09	male	functional
17	52	Larynx SCC (T3N1M0) 07/03	male	functional
18	68	CUP-Syndrom (T0N2bM0) 07/04, OC-SCC (T3N0M0)	male	salvage
19	51	ACC parotid (T3N0M0) 01/07, recurrence 01/08, recurrence 03/08, recurrence 10/08, recurrence 02/09, recurrence 06/09, recurrence 09/09, recurrence 12/09	female	salvage
20	37	OC-SCC (T1N0M0) 09/08, regional recurrence 04/09, regional recurrence 09/09	female	salvage
21	61	Oropharynx-SCC (T2N0M0) 04/09	male	functional

If radiation treatment took place in Heidelberg, radiotherapy was performed in all cases as intensity modulated radiotherapy (IMRT) or at least as a 2D/3-D planned radiotherapy. The applied total doses ranged between 60 and 70.4Gy in a single dose of 1.8 to 2.0Gy. Radiochemotherapy was realized as a combination of 5-FU and cisplatin in the first and last treatment week or of cisplatin once a week during radiotherapy (Table 2).

**Table 2:** Type of reconstruction, peri-operative complications, Karnofsky-performance status (KI or KPS), no data available (n

Number	Indication for surgery	Type of recon-struction	RTX versus RCTX/dose	2/3D versus IMRT	Postoperative complications	Pharyngo-tomy	Karnofsky index preop	Karnofsky index postop
1	hypopharynx recurrence	pec major	RCTX/90,9Gy	2D + 3D Boost	0	1	50	60
2	Fistula after salvage laryngectomy	pec major	RTX/60Gy	2D	0	1	50	60
3	oropharynx recurrence	pec major	RCTX/70Gy	n.d.	1(hemorrhage)	1	40	40
4	oropharynx recurrence	pec major and deltopectoral and trapezius	RCTX/70Gy	2D + 3D Boost	0	1	70	80
5	SCC recurrence temporal bone	lat dorsi	RCTX/70Gy	n.d.	0	0	60	70
6	SCC recurrence temporal bone	lat dorsi	RCTX/70Gy	3D	1(partial flap loss)	0	50	50
7	BSCC recurrence temporal bone	lat dorsi	RTX/ND (>2*60Gy)	n.d.	0	0	60	40
8	Regional recurrence debulking	pec major	RCTX/60Gy	n.d.	0	0	60	70
9	Esophageal stenosis	forearm	RTX/60Gy	n.d.	0	1	60	70
10	Oropharynx SCC	forearm	RTX/62Gy	n.d.	0	1	60	70
11	Larynx SCC	pec major	RTX, RCTX/62Gy+ND (>60Gy)	n.d.	1(fistula)	1	60	60
12	hypopoharynx recurrence	pec major	RTX/60Gy	IMRT	0	1	50	60
13	dysphagia	forearm	RTX/ND (>60Gy)	n.d.	0	1	50	60
14	wound healing complication tracheostoma	deltopectoral	RCTX/70,4Gy	2D	0	0	50	60
15	Nasal dorsum SCC recurrence	forearm	RCTX/60Gy	IMRT	1(fistula)	1	50	60
16	dysphagia due to soft palate defect	forearm	RCTX/65,8Gy	IMRT	0	1	50	60
17	Radiochondronecrosis of larynx	pec major	RCTX/ND (>60Gy)	n.d.	1(wound dehiscence donor site)	1	40	60
18	OC-SCC after CUP	forearm	RTX/ND (>60Gy)	n.d.	1(wound dehiscence donor site	1	60	60
19	parotid recurrence	scapula	RTX/60Gy	IMRT	1(wound dehiscence donor site)	0	60	60
20	regional recurrence	lat dorsi	RCTX/70,4Gy	IMRT	0	0	60	60
21	exposed mandibular bone after RCTX	forearm	RTX/66Gy	IMRT	0	1	80	80

Free and regional flap insetting was in all cases performed with 3.0 Vicryl. In cases of pharyngeal closure the flap was inset into the defect and sutured to the surrounding mucosa. The flaps were NOT just used to reinforce closure as described elsewhere [5]. The medical records of the patients were reviewed and analyzed with respect to tumor stage, treatment history, radiation dose, peri- and postoperative complications, KPS, flap performed, and comorbidities. KPS was determined 3 months after surgery as per literature [11]. Statistical analysis was performed using Kaplan Meier and Fisher's exact test.

Overall 10 out of the 21 patients underwent salvage procedures, 4 were treated for palliation, and 7 patients were operated on to improve function or close fistulas and treat wound healing complications after radiation (Table 1).

**Figure 1:**
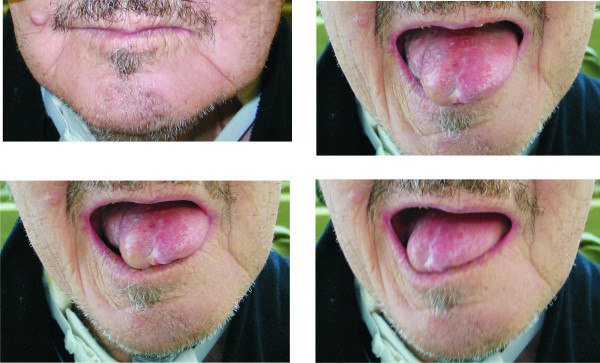
**Reconstruction of the entire anterior tongue (Pt. 18)**. A: For this reconstruction a transoral approach without temporary mandibulotomy was used. B, C, D: Residual mobility preserved through preservation of the base-of-tongue.

**Figure 2:**
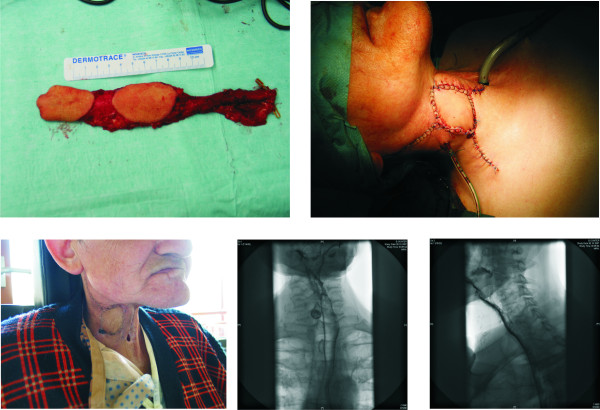
**Reconstruction of a pharyngeal stenosis with a free radial forearm flap (Pt.8)**. A: Flap design with monitor portion. B: Status after flap insetting, monitor visible. C: Postoperative result. D, E: Barium swallow after surgery documenting adequately resolved pharyngeal stenosis after the procedure.

**Figure 3:**
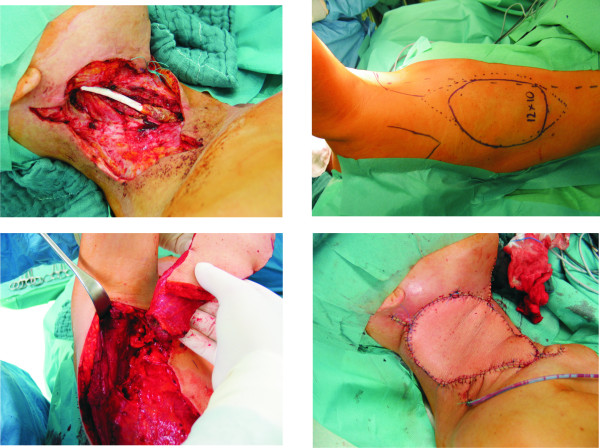
**Reconstruction of the neck with a regional latissimus dorsi flap after radical neck dissection revision and carotid resection with Gore-Tex allotransplant interposition (Pt.20)**. A: Status after resection and Gore-Tex allotransplant interposition. B: Outlines of the flap. C: Harvesting of the flap on the thoracodorsal vessels. D: Status after insetting.

**Figure 4:**
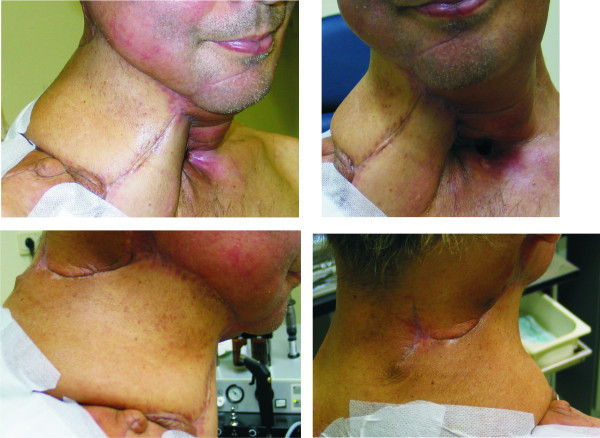
**Reconstruction of the neck after radical extended neck revision with partial pharyngectomy (Pt.4)**. A, B: Reconstruction of the anterior neck with a deltopectoral flap. C, D: Reconstruction of the residual circumference of the neck with a trapezius flap.

Radiation induced wound healing problems are anticipated to occur at the recipient site. Interestingly we observed only 4 recipient site complications (1 hemorrhage from the tracheostomy site, 1 partial flap loss of a regional flap, 1 pharyngeal fistula, 1 fistula in the melolabial region after nasal reconstruction) (19%). We observed three donor site complications (1 wound dehiscence requiring a rotational flap for closure, 1 small wound dehiscences in the scapula flap harvest region and one in the forearm harvest region, requiring no further surgical intervention). In total 7 out of 21 patients had a complication (33%) (Table 2). Of these 7 complications 5 were major complications (24%) and 2 were minor complications (small wound dehiscences that healed via secondary intention and did not require a surgical intervention). Overall 13 pharyngotomies/pharyngeal closures were performed and only 1 fistula was observed (8%). It occurred in a patient who received adjuvant radiation therapy twice up to a cumulative dose of >122Gy. Overall 8 free flaps (7 radial forearm flaps (Figure 1, 2), 1 scapula flap) and 15 regional flaps (8 pectoralis major, 4 latissimus dorsi (Figure 3), 2 deltopectoral (Figure 4a, b), 1 trapezius (Figure 4c, d)) were performed. No free flap failure occurred but one partial flap loss was observed in the group of patients that had received a regional flap with a latissimus dorsi flap. There was no significant association between the incidence of complications and the use of any particular flap. 12 patients received RCTX versus 11 patients that received RTX. All 4 recipient site complications occurred in the RCTX patient population (Table 2). However, this was not found to be statistically significant (Fisher exact, two sided, p = 0.09). Neither age nor preoperative status of comorbidities correlated with the incidence of recipient site or donor site complications within this series of patients.

Median KPS prior to the operation was 60%. Improvement of KPS was observed in 13 out of 21 patients and declined in one patient (Table 2). Looking at the treatment categories 50% of the patients treated for salvage improved with respect to their KPS (5/10). Out of 4 patients treated with palliative intention also 50% improved, one patient had a similar index after treatment but in one patient the index declined. In contrast 80% of the patients treated to improve their functional status improved with respect to their KPS and only one patient had a similar index after the procedure. Both patients treated for a fistula and a post-radiation wound healing complication improved with respect to their KPS.

Within this study 19% (4 patients) of the patients developed recipient site complications. Out of the 4 patients there was only 1 fistula, one tracheostomy bleeding, one partial flap failure of a regional flap and a wound dehiscence requiring additional management. While the fistula an wound dehiscence may be a consequence of radiation-induced recipient site tissue changes, this is less likely the case for the tracheostomy bleeding and complication of a regional flap. We therefore believe that the incidence of wound healing complications that may be a consequence of radiation is only 2 out of 21 patients (9.5%). This suggests that if proper surgical technique is used, operating in the radiated field in the head and neck is likely to be safe. Only 1 patient developed fistula after pharyngeal closure with a regional or free flap. This also compares favorably with published data on fistula formation after salvage laryngectomy, that are reported to be as high as 18% to 29% [5,6]. This data supports in our opinion the conclusion that surgery can be safely performed after radiation once vascularized tissue transfer is used as an adjunct technique.

It is also noteworthy that we did not encounter any flap failures with our free flaps despite having to perform vascular anastomosis in preirradiated and previously operated fields suggesting that free flaps can also be safely performed.

All recipient site complications occurred in patients that were treated with RCTX. This finding however was not statistically significant. This trend however is consistent with published data, indicating a high rate of peri-operative complications after RCTX [2].

In order to assess the potential hazard of an extension of the procedure by a complicated flap surgery on the patient's postoperative performance, we measured the pre- and postoperative KPS based on a chart review. It certainly would have been better to undertake quality-of-life studies in this patient population. However this was not possible due to the retrospective nature of the study. Our data indicate that 62% of our patients had an improved KPS. The preoperative KPS ranged between 40% and 80%, the majority of patients had a KPS between 50% and 70%. A KPS improvement from 50% to 60% indicating to regain the ability to function mostly independent of help in all key areas of live, was observed in 8 out of 21 patients (38%). An improvement from a KPS of 60% to 70% was observed in 4 out of 21 patients (19%) indicating an improvement towards being entirely independent of help without being able to work. The KPS declined in only one patient. The data suggest that 3 months after surgery when the KPS was assessed the patients had recovered very well from surgery despite the extent of the procedure.

In summary our data indicate that surgical treatment of patients after RTX or RCTX is feasible with an acceptable rate of complications, if free or regional flaps are included in the operative strategy.

## Competing interests

The authors declare that they have no competing interests.

## Authors' contributions

CS designed and coordinated the study, participated in the data acquisition and analysis, and helped to draft the manuscript, CB participated in the data acquisition and analysis, MWM and KL both participated in the data acquisition and analysis, and helped to draft the manuscript, ZB and SS and SL participated in the data acquisition and analysis, PKP and PAF helped to coordinate the study and draft the manuscript. All authors read and approved the final manuscript.
